# Parasitological findings in the invasive California kingsnake (*Lampropeltis californiae*) in Gran Canaria, Spain

**DOI:** 10.1017/S0031182021000871

**Published:** 2021-09

**Authors:** Kevin M. Santana-Hernández, Jorge Orós, Simon L. Priestnall, Catalina Monzón-Argüello, Eligia Rodríguez-Ponce

**Affiliations:** 1 Department of Animal Pathology, Faculty of Veterinary Science, University of Las Palmas de Gran Canaria, Las Palmas, Spain; 2 Department of Histology and Pathological Anatomy, Faculty of Veterinary Science, University of Las Palmas de Gran Canaria, Las Palmas, Spain; 3 Department of Pathobiology and Population Sciences, The Royal Veterinary College, Hatfield, UK; 4 EcoAqua University Institute, University of Las Palmas de Gran Canaria, Ctra. de Taliarte, s/n, 35200 Telde, Las Palmas, Spain

**Keywords:** Epidemiology, helminth, histopathology, invasive species, Macaronesia, zoonotic parasites

## Abstract

The California kingsnake (*Lampropeltis californiae)*, native to North America, is a significant threat to the conservation of endemic species in the Spanish Macaronesian island of Gran Canaria. However, its role disseminating potential invasive parasites, such as zoonotic pentastomids, has not been proven. Among its parasitic fauna, only protistans have been documented, in contrast to other *Lampropeltis* spp., which are known to carry pentastomids. Thus, a parasitological study is urgently required. Between 2016 and 2018, a total of 108 snakes were necropsied and stool samples examined. A single snake was infested with *Ophionyssus natricis*, and another individual with *Serpentirhabdias* sp. Only this latter snake presented gross lesions, characterized by granulomatous pneumonia. No Pentastomida were found. By contrast, almost the entire population (98.5%) was infested with larval helminths (three different nematode and two cestode species), characterized by granulomatous gastrointestinal serositis. This suggests the snake poses a ‘dead end’ host for local parasites. Based on these findings, snakes in Gran Canaria carry potential zoonotic mites, which along with *Serpentirhabdias* sp. could represent a threat to endemic lizards. The presence of metazoan parasites and their lesions are reported for the first time in the California kingsnake.

## Graphical abstract

**Figure:**
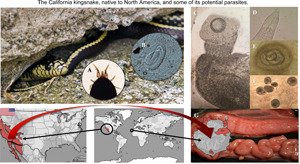

## Introduction

Gran Canaria is an Atlantic island of the Canarian archipelago (27°57′31″N, 15°35′33″W), which belongs to Spanish Macaronesia. The island has a land area of just 1560 km^2^ but reaches 1956 m at the highest point and contains a very diverse ecosystem for its relatively small size. Fifteen endemic species of reptile are found on the Canary Islands, but no snakes.

The introduction of foreign species poses one of the main threats to global biodiversity and ecosystem conservation and its effects are magnified on islands due to ecosystem isolation and high numbers of endemic species or subspecies (Carroll, 2007; Bezerra-Santos *et al*., 2021). This effect has been observed on Gran Canaria where the introduced California kingsnake (*Lampropeltis californiae*) has resulted in a decrease in numbers of the endemic and endangered Gran Canaria giant lizard (*Gallotia stehlini*) in the areas where the snakes have been established the longest – a problem that seems to be getting worse (Gallo-Barneto *et al*., 2016).

**Fig. 1.**
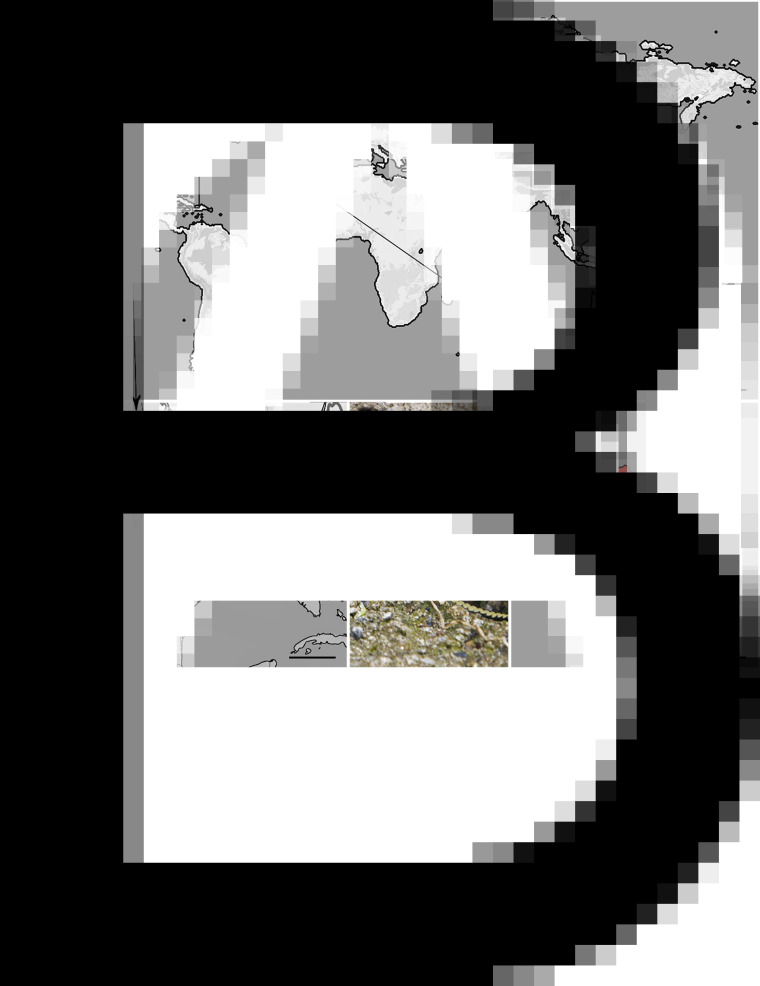
(A) Distribution of California kingsnake in the world. Scale bar = 5000 km. (B) Native locality. Scale bar = 500 km. (C) An adult snake from Gran Canaria. (D) Populations of this invasive reptile in Gran Canaria, Canary Islands, Spain. Scale bar = 10 km. MN, main nucleus; SN, secondary nucleus; TN, tertiary nucleus; FN, fourth nucleus.

The California kingsnake is native from the southwestern USA to the northwestern part of Mexico (Fig. 1) and can be found in a wide range of habitats from forests, rocky areas, coastal, urban and suburban areas, deserts and lakes (Hubbs, 2009). The snake's natural diet is equally wide and includes lizards, geckoes, small turtles and birds, other snakes, skinks and rats among others. This species of snake is generally harmless to humans and has become popular as a pet (Hubbs, 2009). The invasion in Gran Canaria is presumed to be due to accidental escapes or through the release of pet snakes, since at least two of the four populations on the island were established by different groups of captive-bred individuals (Monzón-Argüello *et al*., 2015). The general lack of natural predators, a diet based on at least three endemic reptiles; Gran Canaria giant lizard (*Gallotia stehlini*), Boettger's wall gecko (*Tarentola boettgeri boettgeri*) and Gran Canaria skink (*Chalcides sexlineatus*) and rats, together with its burrowing habits, have firmly established this species as a significant ecological problem (Monzón-Argüello *et al*., 2015).

Invasive species pose a direct threat by preying on native fauna, but they can also harbour new parasites or other pathogens that may cause additional damage to the local environment (Taraschewski, 2006). Moreover, local parasites or pathogens can infect these exotic invaders and may be more harmful to their new (possibly naïve) hosts, than to their native counterparts (Kelehear and Jones, 2010).

Zoonotic parasites, such as *Raillietiella* sp. (Pentastomida) which can cause abdominal lesions in humans due to the migration of their larvae and nymphs, have been reported in *Lampropeltis getula* in the USA (Ali *et al*., 1985; Tappe *et al*., 2016; Mendoza-Roldán *et al*., 2020). Their presence has already been demonstrated in *Gallotia* lizards from the islet of Alegranza (Abreu-Acosta *et al*., 2006), in the eastern Canarian archipelago. If California kingsnakes are able to introduce this pentastomid genus into the island, they could also infect the Gran Canaria giant lizard, as well as potentially humans, and maintain the infection for many generations.

Despite its popularity as a pet, few parasitological studies have been carried out on the California kingsnake, even those kept in zoological collections. Two reports, totalling 11 individuals, describe only protozoan parasites (Van Peenen and Birdwell, 1968; Xiao *et al*., 2004).

This is the first study that describes the lesions caused by helminth parasites in California kingsnakes on Gran Canaria and the trichinoscope (compression plates) is described as a useful tool for the detection of larvae. The possible biological hazard for local fauna, and potentially humans, due to the introduction of foreign parasites is discussed.

## Material and methods

On Gran Canaria, the California kingsnake is located in three distinct nuclei (Fig. 1); the main nucleus (MN), characterized by a wetland area with abundant flora and fauna in the center-east of the island; the secondary nucleus (SN), a very dry and steep location with characteristic Tabaibal-Cardonal flora, in the northwest, and the tertiary nucleus (TN), with similar climatic conditions to the secondary, in the south. A fourth nucleus has recently been identified in the northeast, surrounding the capital city of Las Palmas de Gran Canaria.

Between 2016 and 2018, snakes from the main and secondary nucleus, captured under the extermination plan approved by the Canary Islands government, were necropsied at the Faculty of Veterinary Science of the University of Las Palmas de Gran Canaria following standard procedures (Farris *et al*., 2013). Due to the few individuals captured, in both cases less than five, the tertiary and fourth nuclei were not included in this study. Biometrical parameters of weight, length, fat weight and sex were recorded. The infested tissues from the animals were fixed in 10% neutral-buffered formalin and routinely processed to paraffin blocks, serially sectioned at 5 *μ*m and stained with hematoxylin and eosin.

Routine fecal examinations were performed following standard methods (Zajac and Conboy, 2012) and detailed notes made on the appearance and number of any gross pathological changes. Gross lesions were further assessed microscopically using compression plates (trichinoscopy). The results are given as prevalence, mean intensity and abundance (Bush *et al*., 1997).

The parasites were extracted with the help of mounting needles and identified in temporary mounts with saline solution following the keys cited in the bibliography for cestodes (Witenberg, 1932; Joyeux and Baer, 1936; Ryšavý, 1973; Jones, 1994), nematodes (Chitwood and Wehr, 1934; Moravec *et al*., 1987; Anderson, 2000; Willmot and Chabaud, 2009; Kelehear and Jones, 2010) and Acari (Evans, 1955; Lindquist *et al*., 2009; Moraza *et al*., 2009).

Statistical analysis was performed with SPSS v. 24.0 (IBM SPSS Corp., Chicago, Illinois, USA). The parasite number variation was tested using the parametric Student *t*-test for each population, the correlations between both weight/length and parasite number were assessed by means of Pearson or Spearman correlation tests, after testing for normality. *P* values <0.05 were considered to be statistically significant.

## Results

### Biological sample data: ecological indices and correlations

In total, 108 snakes were examined, divided into two groups: Group A; the first 44 samples collected, in which coprological and postmortem examinations were performed using a stereoscopic microscope, and group B; the following 64 samples, in which the postmortem examinations were enhanced with the aid of a trichinoscope (Table 1). The biometrical parameters and sex by nuclei are represented in Table 2.

**Table 1.** Ecological indices for the larval parasites found in the main (MN) and secondary nucleus (SN)

	Group A *n* = 44	Group B *n* = 64		
	Prevalence (%)	Prevalence (%)	Mean intensity	Mean abundance
MN				
Helminths	55.2	97.0	81.1 ± 118.8	78.7 ± 117.7
Nematodes	^a^	69.7	62.7 ± 133.0	43.7 ± 114.0
Cestodes	^a^	81.5	42.7 ± 49.5	34.9 ± 47.7
SN				
Helminths	80.0	100.0	203.9 ± 294.9	203.9 ± 294.9
Nematodes	^a^	90.0	186.0 ± 306.5	167.4 ± 295.8
Cestodes	^a^	83.3	43.8 ± 33.2	36.5 ± 34.4

a Unable to calculate.

**Table 2.** Sample biometrical parameters

	Females	Males	ML (cm)	MW (g)	FW (g)	F%	Total
MN	33	26	88.3 ± 3.1	167.0 ± 15.9	14.1 ± 2.4	8.7 ± 1.9	59
SN	16	28	89.4 ± 3.7	163.9 ± 15.9	9.4 ± 1.2	5.7 ± 0.5	44
UN							5

ML, mean length; MW, mean weight; FW, mean fat weight; *F*%, mean fat percentage of the body weight; MN, main nucleus; SN, secondary nucleus; UN, undetermined sex and/or nuclei.

**Fig. 2.**
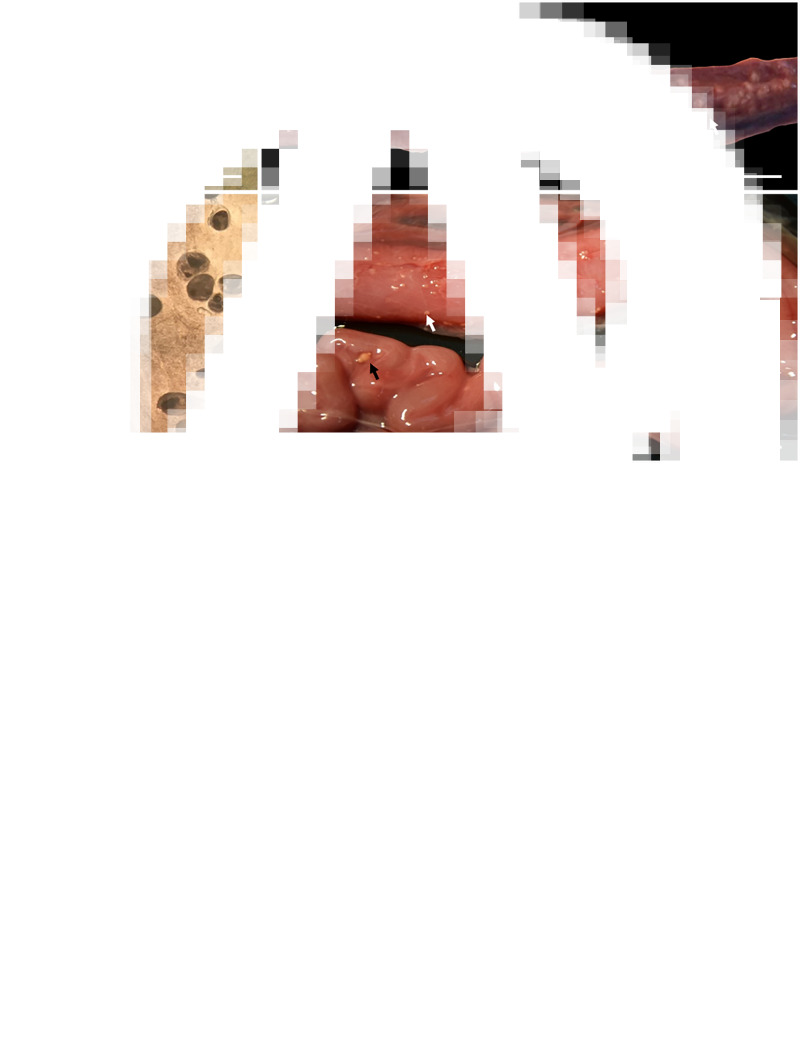
(A) Larval nematode encysted in the gastric wall of a snake. Scale bar = 200 *μ*m. (B) Gross appearance of a partly opened lung, showing multiple granulomas (white arrows). Scale bar = 10 mm. (C) Larval tapeworms encysted in the intestinal wall. Scale bar = 1 mm. (D) Gross image of granulomas on the gastric (top, white arrows) and intestinal (bottom, black arrow) serosa. Scale bar = 10 mm.

In group A snakes, assessed macroscopically, 59.1% had parasitic cysts, with 25.0% containing cysts in the stomach and 45.5% in the small intestine (Figs 2A and C). 80.0% of SN snakes harboured these cysts and 55.2% from the MN.

By contrast, group B, tested by trichinoscopy, revealed a prevalence of 98.4% with cysts – 100% of SN snakes and 96.9% of those from the MN. These cysts microscopically corresponded to cestode cysticercoids and nematode larvae. In summary, almost 30% more cysts were observed with trichinoscopy than by gross examination alone.

No significant differences in parasitic burden or prevalence were found between sex, colour and line patterns.

The prevalence of helminths was estimated using group B. No differences in cestode prevalence were observed between the MN and the SN (80.6 and 83.3% respectively). However, nematode prevalence was markedly different; 90.0% in the SN and 69.7% in the MN.

The two nuclei showed a significant difference in the average number of helminths per individual snake (Student *t*-test, *P* = 0.008), but there was no significant difference in numbers of cestodes between the two nuclei (*P* = 0.307; Mann–Whitney's *U* test).

In terms of intensity and abundance, the SN showed the highest mean nematode intensity and abundance, and the highest individual infestation (186 ± 306.5; 167.4 ± 296 and 492 larvae respectively). However, there were few differences between the mean intensity and abundance of tapeworms in the two nuclei. Highest tapeworm intensity was found in the MN.

The anatomic distribution of the helminths was not equal; nematodes were concentrated in the stomach, reducing in prevalence and abundance from stomach to large intestine (73.4% stomach, 67.2% small intestine and 42.2% large intestine). Similarly, cestodes were concentrated in the small intestine and reduced in numbers in the large intestine, with no records of cysticercoids in the stomach (81.0% small intestine, 51.6% large intestine and 0% stomach). This anatomic distribution was recorded in both populations studied. No correlations were found between the number of cestodes and the length of the snakes (*ρ* = −0.144, *P* = 0.448; Spearman *ρ* test for the SN and *ρ* = −0.192, *P* = 0.285; Spearman *ρ* test for the MN); however, the number of nematodes showed a positive correlation with the length of the snakes (*P* = 0.022 for the SN and *P* = 0.008 for the MN).

No significant correlations were found between the fat percentage of the body weight or fat weight with parasitic burden.

### Coprological examination

**Fig. 3.**
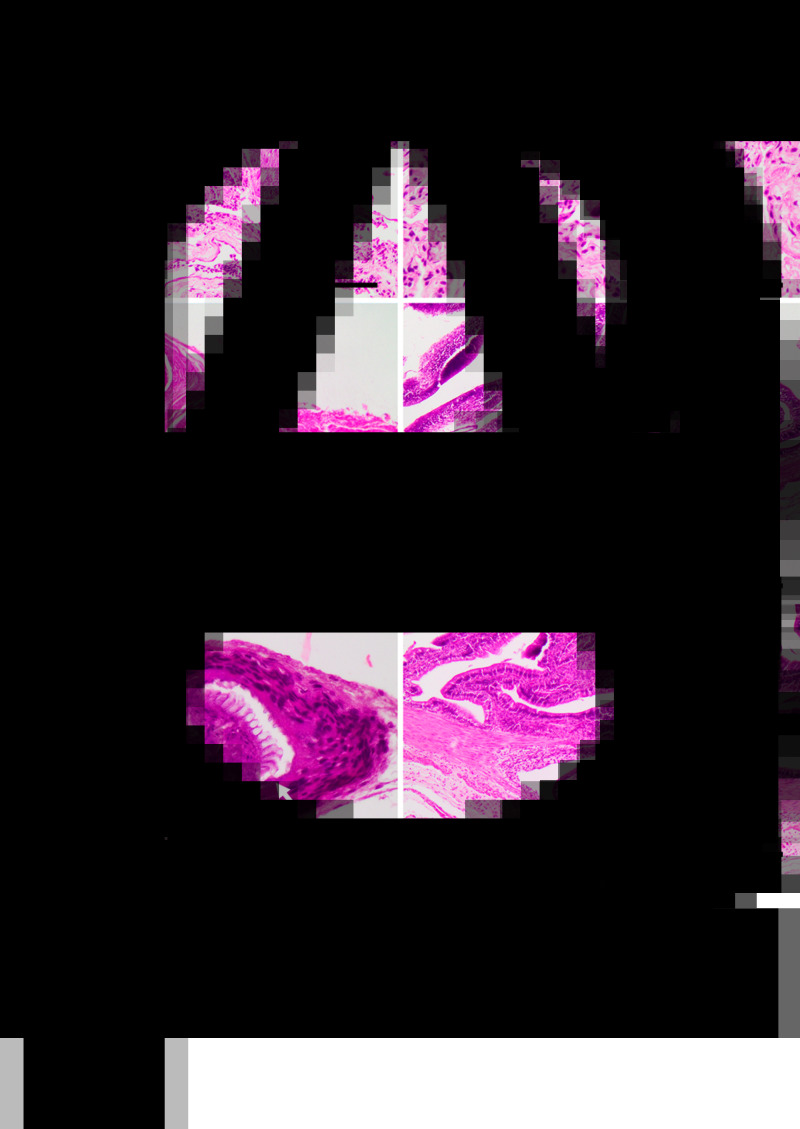
Histological sections showing, (A) lung with adults (arrow heads) and larvae (arrow) of *Serpentirhabdias* sp. H&E. Scale bar = 100 *μ*m. (B) Lung with atelectasis and mild suppurative inflammation. Note the longitudinal section of the head of a *Serpentirhabdias* larva (arrow). H&E. Scale bar = 40 *μ*m. (C) Lung granulomas. H&E. Scale bar = 200 *μ*m. (D): A chronic-active granuloma, with intralesional dead nematode, on the intestinal serosa (arrow). H&E. Scale bar = 200 *μ*m. (E) A chronic granuloma, containing a dead tapeworm surrounded by a wall of fibrocytes. Note the preserved rostellum (arrow). H&E. Scale bar = 40 *μ*m. (F) A cystic granuloma in the muscular layer of the intestine containing a cysticercoid. H&E. Scale bar = 200 *μ*m.

Just one snake out of the 44 (group A) contained parasite eggs and larvae in its feces compatible with rhabditid nematodes which, in addition to the observation of granulomatous lesions in the lung (Figs 2B, 3A and B), and no adult parasites in the intestine, confirms the diagnosis of rhabdiasid lungworm eggs and larvae, compatible with the genus *Serpentirhabdias*.

### Adult parasites

**Fig. 4.**
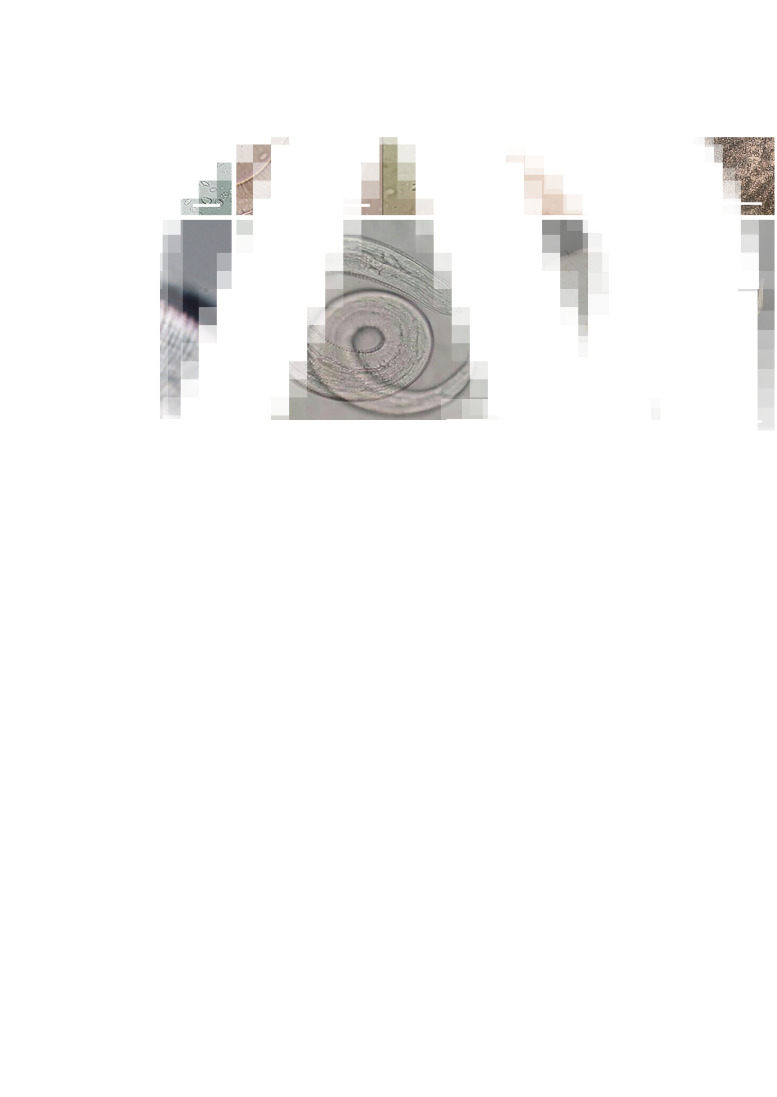
(A) Rostellum in apical view, note the five rows of hooks. Scale bar = 50 *μ*m of *Diplopylidium acanthotetra*. (B) Morphological type No. 1 of larval spirurids, possible Spirocercidae. Inset: posterior end. Note the two papillae on the anterior end, and the tuft of digitations on the tail. Scale bar (left) = 50 *μ*m, scale bar (right) = 10 *μ*m. (C) Cysticercoid of *Diplopylidium acanthotetra.* Scale bar = 200 *μ*m. (D) Morphological type No. 2 of larval spirurids, possible Acuarinae. Note the two papillae and the straight reticular cordon. Scale bar = 50 *μ*m. (E) Morphological type No. 3 of larval spirurids, possible Physalopteroidea. Scale bar = 50 *μ*m. (F) Cleared specimen of *Ophionyssus naticis*. Scale bar = 200 *μ*m.

Just two specimens out of the 108 snakes showed adult parasites, one specimen from each group (A and B). From group A, the snake passing parasite eggs in its feces was found infested with *Serpentirhabdias* lungworms. From group B, one snake harboured 19 Mesostigmatid skin mites of the family Macronyssidae, in various life stages, compatible with *Ophionyssus natricis* (Fig. 4F).

### Identification of the larval helminths

The metacestodes (Figs 2C, 4A and C) found were characterized as a solid larval form, with 4 suckers and 4–5 rows of taenioid and rose thorn hooks which identifies them as two species of the genus *Diplopylidium*, *D. acanthotetra* (70.3% prevalence) and *D. nölleri* (21.9% prevalence). Several specimens of both species were deposited at the Parasites and Vectors collection of the Natural History Museum of London (accession numbers: NHMUK.2020.2.12.1-2 and NHMUK.2020.2.12.3).

Nematodes were characterized based on the divided oesophagus (muscular and glandular) and the three morphological types found correspond to the order Spirurida and, based on other morphological features, possibly to three superfamilies: Spiruroidea (Type 1), Acuarioidea (Type 2) and Physalopteroidea (Type 3).

The first and most common helminth (Fig. 4B) (45.3% prevalence) was characterized by two prominent papillae at the anterior end and thin lateral wings that begin posterior to the nerve ring, up to a few micrometres before the cloaca. These also possess a tuft of finger-shaped papillae at the posterior end. This description matches several larvae from genera of the same taxa: superfamily Spiruroidea, family Spirocercidae.

In the second type (Fig. 4D) (35.9% prevalence), there were two pseudo-labia at the anterior end, accompanied by four non-recurrent or anastomosed reticular cordons and two monocuspic cervical papillae. At the posterior end, no papillae or other ornaments were found. These characteristics match those described for larvae from the superfamily Acuarioidea, subfamily Acuarinae, which are the only ones in which larvae have cephalic cordons. Finally, the third (Fig. 4E) and smallest morphological type (4.7% prevalence) was characterized by two trilobed lips, a posterior end without papillae and thin lateral alae. These larvae likely belong to the superfamily Physalopteroidea.

### Gross and microscopic findings

One snake presented with diffuse red discolouration of the lung with numerous, multifocal to coalescing white, round nodules, 0.5–2 mm diameter, affecting around 70% of the cranial lung parenchyma (Fig. 2B).

Microscopically, these lesions corresponded to multifocal mild verminous pneumonia composed of a mild infiltration of heterophils, macrophages and lymphocytes, as well as a small amount of cellular debris, fibrin and oedema surrounding adult and larval nematodes inside faveolar spaces. Mild pulmonary congestion as well as faveolar atelectasis were also observed (Figs 3B and A). In addition, occasional pleural granulomas with intralesional bacteria were observed (Fig. 3C).

The parasites were characterized by the presence of a body cavity and an external eosinophilic cuticle, surrounding an inner layer of degenerated platymyarian muscle fibres, a digestive tract and a reproductive structure compatible with ovary (Fig. 3A). Also present, predominantly within faveolar walls and the interstitium, were often highly cellular and basophilic larval forms. On sagittal sections, a single-cell digestive tract was observed – compatible with early larval stages (L1–L2).

In total, 98.4% of snakes presented with moderate to severe multifocal white nodular lesions from 0.7 to 5 mm in diameter on the serosal surface of the intestine and stomach, and coelomic wall (Fig. 2D). Microscopically, these lesions were present in various stages of maturity (Figs 3D and E) and were located mostly in the serosal and muscular layer of the intestine (Fig. 3F) and coelomic wall (Fig. 3E). The lesions were microscopically characterized as cystic granulomas, composed of an outer layer of fibroblasts, fibrocytes and collagen surrounded by several layers of compacted macrophages and few lymphocytes and heterophils, surrounding a parasitic structure that floated in an unstained fluid. These parasites (Cestoda) were characterized by the presence of an outer thick eosinophilic tegumentary layer, lack of body cavity, and in its place a vacuolated space without digestive system, presence of armed scolex and suckers, lack of reproductive organs and presence of calcareous corpuscles.

Non-cystic granulomas were also found containing cysticercoids, with a well-preserved rostellum, surrounded by numerous macrophages, lymphocytes, fibroblasts and fibrocytes, or with more active inflammation including the presence of heterophils inside the granulomas (Figs 3D and E). Associated with these latter areas of inflammation were degenerated nematode larvae (Fig. 3D).

## Discussion

Here the first record of two *Diplopylidium* species and three larval nematode morphological types in the California kingsnake are described. These findings are consistent with local parasites infecting an invasive species, which would act as a ‘dead-end’ paratenic host, since no predators are described for this snake in Gran Canaria.

Larval helminth cysts from reptiles, and snakes specifically, have been reported previously in the literature; however, just *Mesocestoides* tetrathyridia and *Macracanthorhynchus* cystacanths, have been described in a *Lampropeltis* sp. snake (Elkins and Nickol, 1983; Jacobson, 2007).

Only larvae from members of the superfamilies Spiruroidea, Acuarioidea and Physalopteroidea have been commonly described parasitizing reptiles (Anderson, 2000; Criscione and Font, 2001; Goldberg and Bursey, 2001; Santos *et al*., 2006). Specifically; *Physocephalus*, *Ascarops* and *Spirocerca lupi*, from Spirocercidae (Moravec *et al*., 1987; Goldberg and Bursey, 1988; Goldberg *et al*., 1994), Acuarinae *gen*. from Acuarioidea (Roca, 1985; Moravec *et al*., 1987) and *Physaloptera*, from Physalopteroidea (Widmer, 1970).

Regardless of the parasitic species, the burden and prevalence found in the Gran Canaria snake population (98.4%) is considerably higher than that reported for larval parasites from other reptiles in Spain, and indeed North America (usually less than 2%) (Widmer, 1967; Roca, 1985; Martín *et al*., 2003; Santos *et al*., 2006; Goldberg *et al*., 2013; Davis *et al*., 2016). Furthermore, there are no records of *Diplopylidium* species in North American fauna (Criscione and Font, 2001; Goldberg and Bursey, 2001; Yildirimhan *et al*., 2005; Goldberg *et al*., 2013; Davis *et al*., 2016; McAllister and Bursey, 2016). This difference could be due to the use of macroscopic examination during necropsy, instead of using compression plates (trichinoscopy) to see through the organ. In fact, the data obtained using the two techniques in this study differ by almost 30%, with the more sensitive utilization of trichinoscopy being developed through research on larval parasitic forms of *Trichinella*.

Due to the lack of studies in the California kingsnake, it is not possible to compare the epidemiological results here with the native American populations of these snakes. In the Canary Islands, a maximum prevalence of 21.7% for *D. nölleri* and 2.2% for *D. acanthotetra* (Roca *et al*., 1999) is reported in the endemic gecko species described as natural hosts for the tapeworm species. Meanwhile in its invasive host the general prevalence was 25.8 and 76.7%, respectively. Moreover, the mean intensity registered for *Diplopylidium* species in endemic geckoes was also lower, reaching a maximum of 37.5 cysticercoids for *D. nölleri* from the Boettger's wall gecko (*T. boettgeri*) in Gran Canaria, *vs* 43.8 larvae in the snakes. In addition, the anatomic distribution of the cestodes was similar in the two hosts; in both species on the intestinal serosa, but in the case of geckoes, they were also found on the liver surface (Roca *et al*., 1987). These findings could mean that *Diplopylidium* cysticercoids are being transferred from geckoes to the snakes. The only suitable definitive carnivore hosts on the island are cats and dogs, with only *Diplopylidium acanthotetra* described in cats from the island of Tenerife (Sánchez, 2013).

There is only one report of larval nematodes, which match the morphology described in this study as ‘Type 1’, parasitizing the endemic Boettger's lizard (*Gallotia caesaris*) on the island of El Hierro (Martín *et al*., 2003). In that study, they reported a mean intensity of 2 larvae and a prevalence of 2.5%, which is significantly lower in comparison with the mean intensity of 186 larvae and 90% prevalence found in these kingsnakes. Apart from the report from *G. caesaris* from El Hierro, no other larval nematodes have been recorded in vertebrate hosts which can be eaten by the snakes in Gran Canaria, thus the previous paratenic host remains unknown.

Furthermore, it has been demonstrated that geckoes can harbour larval spirurids of the three morphological types described in this study (Criscione and Font, 2001; McAllister and Bursey, 2016), therefore despite the negative results found in Gran Canaria populations, Boettger's wall gecko would be a suitable previous paratenic host for these nematodes.

*Ophionyssus natricis* is a widely distributed mite that represents a potential zoonotic risk, predominantly in suburban areas, where the wildlife–human interface shrinks. There are no reported cases of human infestation from Gran Canaria, however, with concentrated sampling effort of habitations within the larger nuclei, its presence may be demonstrated. In addition to dermatitis in humans (Schultz, 1975), other reptiles can be infested with this mite (Norval *et al*., 2020) which can also act as a vector for *Aeromonas* sp. (Wozniak and DeNardo, 2000), leading to fatal disease in certain cases. Hence, the possible spillover of these mites, as well as *Serpetirhabdias* sp., to local fauna, requires further research.

In addition to the ecosystem damage caused by predation of native fauna and competition with other native predators for food, these snakes can be considered ‘dead-end’ paratenic hosts. Most of the parasites found here will not be able to reach their respective definitive hosts, such as birds of prey, and thus are disrupting natural life cycles in the island. For example, once the population of geckoes decreases, the transmission of parasites, such as acuariid nematodes, will likely be affected, since these are transmitted to birds of prey by eating geckoes (as paratenic hosts) rather than consuming terrestrial isopods or other arthropods (intermediate hosts). This is a rather less obvious, but no less important, consequence of the presence of invasive snakes which will be contributing not only to the extinction of geckoes and other native reptiles, but also to their respective parasites of which several are endemic to the Canary Islands, e.g. *Thelandros filiformis*, *Alaeuris stehlini*, *Sarcocystis stehlini*, *Ophionyssus setosus*.

There was a positive correlation between nematode burden and the age of the snake, a correlation that did not exist for *Diplopylidium* sp. When an animal is exposed to a new parasite, the immune response is usually more severe than would occur in response to a parasite with which it had had co-evolved (Kelehear and Jones, 2010). No records of *Diplopylidium* have been found for North American fauna, unlike larval spirurids, therefore in its natural habitat, the California kingsnake would not be exposed to *Diplopylidium.* An increased immune response to new parasites could mean that the California kingsnake more efficiently controls tapeworms, rather than accumulating them during life as with nematodes, which would explain the positive correlation between longevity and parasite burden. Moreover, this could also explain how, if the two parasitic groups have the same donor host, such as the Boettger's wall gecko, that a correlation could exist for just one.

Rhabdiasid lungworms are considered common parasites of amphibians and reptiles (Anderson, 2000); however, their prevalence in North American snakes is very low. Only single cases of rhabdiasid-induced pneumonias, with limited gross and histological descriptions, are reported in snakes from various countries (Jacobson, 2007; Langford, 2010; Mihalca *et al*., 2010; Goldberg *et al*., 2013; Davis *et al*., 2016).

Of the eight genera of Rhabdiasid nematodes which can infest reptiles, only one has been described in *Lampropeltis* spp. snakes, with two species: *Serpentirhabdias fuscovenosa* and *S. eustreptos* (Langford, 2010).

The low prevalence of *Serpentirhabdias* in kingsnakes in Gran Canaria could be explained by the relatively infrequent exposure of the snakes to their own feces in the environment, and their captive-bred origin. These first feral, presumed dewormed, animals had been released into an environment lacking existing snake parasites or natural paratenic hosts.

Parasitic pneumonias in snakes are reported with variable severity, from suppurative pneumonias with clear clinical signs and death (Jacobson, 2007), to those with only mild microscopic changes (Santos *et al*., 2008).

Infective larvae of *Serpentirhabdias* sp. can inoculate soil bacteria, carried inside their intestines, within the snake lung, resulting in a significant secondary bacterial pneumonia with prominent gross lesions (Santos *et al*., 2008). Various Gram-negative bacteria such as *Proteus* sp. and *Pseudomonas* sp. (Hilf *et al*., 1990; Santos *et al*., 2008) have been isolated from parasitized animals, likely acting as opportunistic agents, rather than the primary pathogen. Thus, the lung granulomas found in this study are possibly caused by inoculated soil bacteria, through indirect parasite damage.

*Physaloptera* sp. nematodes are described producing gastric lesions *via* larval penetration in a prairie rattlesnake (*Crotalus viridis)* in the USA (Widmer, 1970). In contrast, in this study, encysted larvae are described, with the small and large intestine representing a new anatomic location for these parasites. In addition, similar lesions to this report have been described for larval spirurids in several reptiles and amphibians (Goldberg and Bursey, 1988; McAllister *et al*., 1993; Goldberg *et al*., 1994).
